# Meta-CD: a metagenomic sequencing coverage and depth calculator for target species

**DOI:** 10.1128/mra.00811-26

**Published:** 2026-09-04

**Authors:** Callie Claiborne, Zhe Lyu

**Affiliations:** 1 Department of Plant and Microbial Biology, North Carolina State University 242504, Raleigh, North Carolina, USA; University of Michigan, Ann Arbor, Michigan, USA

**Keywords:** metagenomics, microbiome, software, target species

## Abstract

**Meta**genomic **C**overage and **D**epth Calculator (Meta-CD) is a convenient, biologist-friendly tool for determining coverage and depth to enhance taxonomic detection, functional profiling, and metagenome-assembled genome (MAG) recovery in metagenomics. It supports experimental design and post-sequencing analysis, modeling how genome size, relative abundance, sequencing depth, and DNA quantity influence detection of target species.

## ANNOUNCEMENT

Accurately determining whether a target microbial species can be detected in a shotgun metagenomic sequencing study requires understanding how biological and sequencing parameters interact (1–3). **Meta**genomic **C**overage and **D**epth Calculator (Meta-CD) determines coverage and depth for targeted metagenomic applications, where the abundance of the target organism is known, estimated, or experimentally controlled. Such contexts include post-sequencing data sets, enrichment/selective cultures, and mock communities, where species-specific coverage predictions are biologically meaningful.

**Fig 1:**
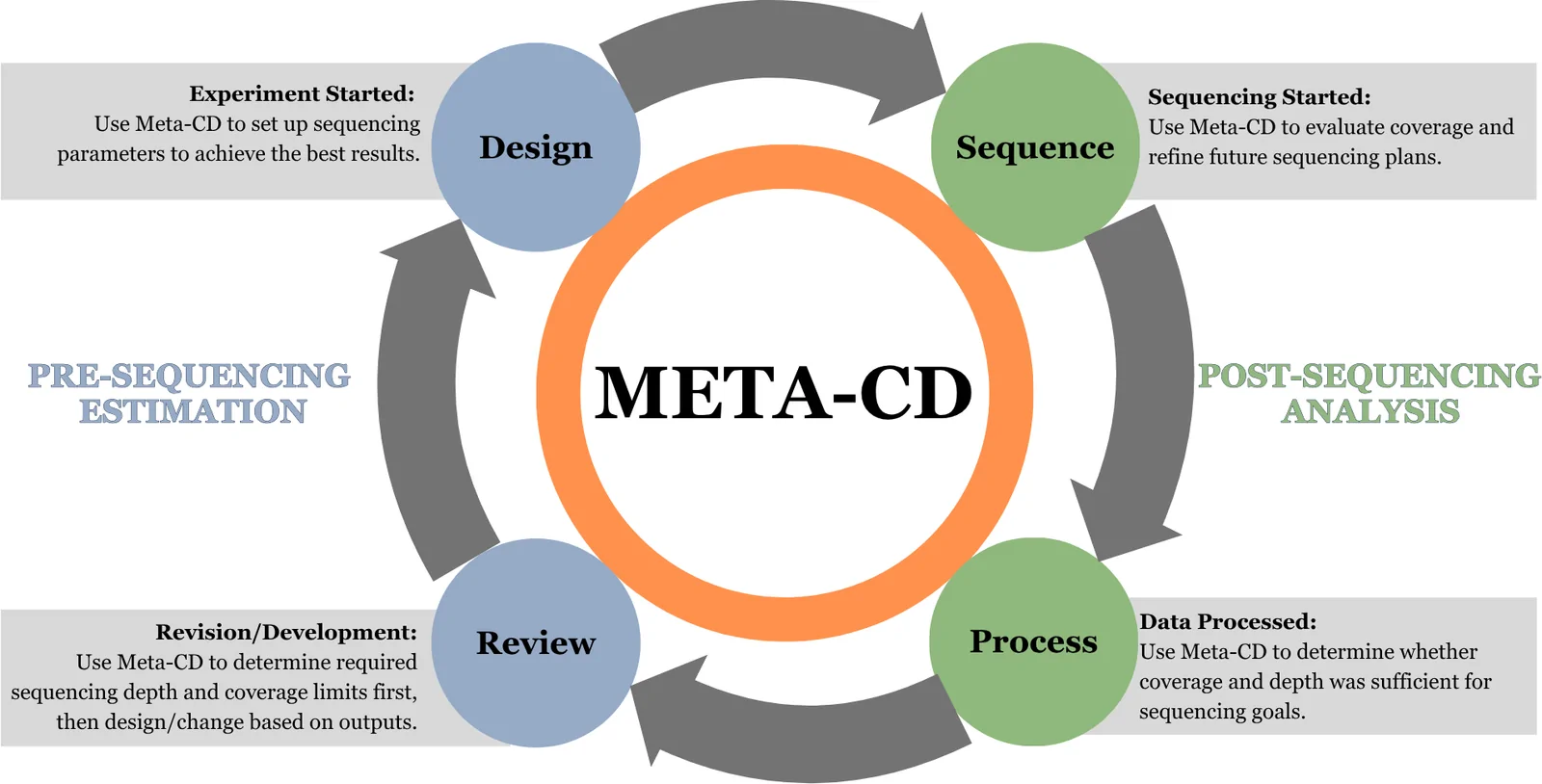
Meta-CD integration into metagenomic study: Meta-CD supports both pre-sequencing planning and post-sequencing evaluation. Users may enter the cycle at any stage (Design, Sequence, Process, or Review) of a metagenomic study.

In these settings, genome size and relative abundance shape the biological context, while sequencing depth and DNA input constrain effective recovery of biological information. However, this interaction is poorly modeled, hindering biologically motivated experimental design and sequencing analysis (2–4). Consequently, researchers lack a simple way to assess the detectability of a target species under biologically relevant sequencing constraints (2, 5). Beyond taxonomic profiling, additional parameters must be integrated to inform functional pathway analysis and metagenome-assembled genome (MAG) recovery (6). Meta-CD addresses this gap by integrating biological and sequencing parameters to support two applications: (i) Pre-Sequencing Estimation—estimating sequencing depth required to reach a target coverage threshold; and (ii) Post-Sequencing Analysis—estimating species-specific coverage given sequencing depth and relative abundance (Fig. 1). Users may utilize Meta-CD at any stage of a metagenomic study to refine experimental planning or evaluate sequencing outcomes.

Meta-CD implements a deterministic model and integrates user-defined biological and sequencing parameters, namely, relative abundance, genome size, sequencing depth, and DNA input quantity, to estimate species-specific coverage (1, 2, 4, 7). It calculates the expected number of bases originating from a species and converts this into per-base coverage (1, 3, 7). When nominal sequencing depth exceeds the number of unique DNA base pairs available in a sample, Meta-CD applies a DNA-quantity-constrained correction to maintain biologically relevant coverage estimates (3, 4, 8, 9). Users may select the coverage threshold from 1 to 30× to support applications ranging from taxonomic detection and functional profiling to high-confidence variant calling for MAG recovery (2, 6).

Meta-CD is available as a browser-based application written in HTML, CSS, and JavaScript. All computations occur locally in the user’s browser. The interface provides real-time updates, interactive inputs, and two visual reference components: a MAG Recovery Potential Table and a Coverage Estimation Table summarizing expected coverage across sequencing depth and relative abundance. The full source code is publicly available with version control, documentation, an MIT license, and an issue tracker. No registration or external data sets are required.

All calculations performed by Meta-CD follow the mathematical framework summarized in Table 1, which integrates sequencing depth, relative abundance, genome size, and DNA quantity to generate each output (1, 7, 8). These equations underpin all reported values and ensure transparent, reproducible interpretation of species-level coverage estimates (3). Results have been validated using the MBARC-26 mock community, a defined mixture of 26 bacterial and archaeal genomes, with known genome sizes, molarity, genome copy numbers, and sequencing representation (10).

**TABLE 1:** Explanation of calculations performed by Meta-CD^*a*^

Output	Formula/calculation	Explanation	Ref
D_community_ (base conversion)	$\begin{array}{c} D_{community}\;(bases)=\\ D_{community}\;(Gb)\times 10^{9} \end{array}$	Total sequencing depth converted from gigabases to bases	(7)
Maximum unique depth (DNA-limited)	$D_{\max}=DNA_{ng}$	Approximation that 1 ng DNA yields ~1 Gbp of the unique sequences	(4, 8)
Species-specific sequencing bases	$B_{species}=D_{community}\times 10^{9}\times A$	Total number of DNA bases sequenced from target species. Sequencing depth (in Gb) is converted to bases and multiplied by the relative abundance of the target species	(3, 7)
Coverage from sequencing depth	$C=\frac{D_{effective}\times 1000\times A}{G}$	Expected average coverage for a genome of size G at abundance A, using effective depth adjusted for DNA-input constraint	(7, 11)
Effective sequencing depth (DNA-limited)	$D_{effective}=\min(D_{total},D_{\max})$	If DNA input constrains library complexity, the effective depth is capped at *D_max_* (1 ng ≈ 1 Gbp DNA base pairs)	(4, 8)
Required sequencing depth	$D_{required}=\frac{C_{target}\times G}{1000\times A}$	Sequencing depth needed to reach target coverage for a species at a given relative abundance	(3, 7, 11)
Minimum detectable relative abundance	$A_{\min}=\frac{C_{target}\times G}{1000\times D_{effective}}$	Lowest relative abundance at which the species reaches the target coverage under the available effective depth.	(3, 7, 11)
Naive coverage (no DNA limit)	$C_{naive}=\frac{D_{total}\times 1000\times A}{G}$	Coverage assuming unlimited DNA input	(3, 7)
MAG recovery coverage	$C=\frac{D_{effective}\times 1000\times A}{G}$	Same formula as coverage; interpreted using MAG thresholds (1×, 5×, 10×, and 20×)	(6, 12)
Coverage estimation table values	Computed using *C_effective_* across a matrix of depths and relative abundances	The table shows expected coverage for the pre-computed matrix automatically adjusted for genome size and DNA quantity	(7, 8, 10)

^*a*^ Mathematical calculations used in Meta-CD to compute species-specific sequencing bases, coverage, detection limits, and required sequencing depth. Variables are defined as follows: D_community_ (total sequencing depth, Gb), D_effective_ (effective sequencing depth adjusted for DNA-input constraints), D_max_ (maximum sequencing depth constrained by DNA quantity), G (genome size, Mb), A (relative abundance as a fraction), A_min_ (minimum relative abundance required to reach target coverage), C (coverage), C_target_ (user-defined target coverage), B_species_ (bases sequenced from the target species), and C_effective_ (effective coverage adjusted for DNA-input constraint). All calculations follow the standard coverage equation and incorporate DNA-quantity constraints where applicable.

In summary, Meta-CD predicts required sequencing depth for experimental design (pre-sequencing estimation) and the likelihood of taxonomic detection, functional profiling, and MAG recovery for a given metagenomic data set (post-sequencing analysis). We hope Meta-CD will help researchers make quantitative and biologically informed decisions throughout the cycle of metagenomic studies.

## Data Availability

Meta-CD is available as a public web application at https://cysteger.github.io/Meta-CD/. The full source code, including all functions, documentation, and interface components, is openly accessible in a GitHub repository (https://github.com/cysteger/Meta-CD, DOI: https://doi.org/10.5281/zenodo.21936365) under an MIT license. The repository includes installation instructions, a README, a complete test suite with example inputs and expected outputs, and an issue tracker that is readable without registration. Users may submit issues or pull requests without manual approval. Meta-CD runs entirely in the browser and does not require external data or user registration.

## EDITOR'S NOTE

**Ed. Note**: This article was handled by Pat Schloss, who acted as a guest editor in consultation with the Editor in Chief, Irene Newton. Dr. Schloss's role as the guest editor of this article is outside of his duties as the Chair of the Journals Committee.

## References

[1] Nayfach S, Pollard KS. 2015. Average genome size estimation improves comparative metagenomics and sheds light on the functional ecology of the human microbiome. Genome Biol 16:51. doi:10.1186/s13059-015-0611-7 PMC4389708 25853934

[2] Treichel NS, Pauvert C, Séneca J, Pjevac P, Berry D, Penders J, Hitch TCA, Clavel T. 2026. Benchmarking of shotgun sequencing depth reveals the potential and limitations of shallow metagenomics and strain-level analysis. Nat Microbiol 11:1233–1244. doi:10.1038/s41564-026-02334-2 PMC13171431 42014453

[3] Nayfach S, Pollard KS. 2016. Toward accurate and quantitative comparative metagenomics. Cell 166:1103–1116. doi:10.1016/j.cell.2016.08.007 PMC5080976 27565341

[4] Daley T, Smith AD. 2013. Predicting the molecular complexity of sequencing libraries. Nat Methods 10:325–327. doi:10.1038/nmeth.2375 PMC3612374 23435259

[5] Tremblay J, Schreiber L, Greer CW. 2022. High-resolution shotgun metagenomics: the more data, the better? Brief Bioinform 23:bbac443. doi:10.1093/bib/bbac443 36352504

[6] Parks DH, Rinke C, Chuvochina M, Chaumeil P-A, Woodcroft BJ, Evans PN, Hugenholtz P, Tyson GW. 2017. Recovery of nearly 8,000 metagenome-assembled genomes substantially expands the tree of life. Nat Microbiol 2:1533–1542. doi:10.1038/s41564-017-0012-7 28894102

[7] Lander ES, Waterman MS. 1988. Genomic mapping by fingerprinting random clones: a mathematical analysis. Genomics 2:231–239. doi:10.1016/0888-7543(88)90007-9 3294162

[8] McNulty SN, Mann PR, Robinson JA, Duncavage EJ, Pfeifer JD. 2020. Impact of reducing DNA input on next-generation sequencing library complexity and variant detection. J Mol Diagn 22:720–727. doi:10.1016/j.jmoldx.2020.02.003 32142899

[9] Deng C, Daley T, Calabrese P, Ren J, Smith AD. 2020. Predicting the number of bases to attain sufficient coverage in high-throughput sequencing experiments. J Comput Biol 27:1130–1143. doi:10.1089/cmb.2019.0264 PMC7398442 31725321

[10] Singer E, Andreopoulos B, Bowers RM, Lee J, Deshpande S, Chiniquy J, Ciobanu D, Klenk H-P, Zane M, Daum C, Clum A, Cheng J-F, Copeland A, Woyke T. 2016. Next generation sequencing data of a defined microbial mock community. Sci Data 3:160081. doi:10.1038/sdata.2016.81 PMC5037974 27673566

[11] Wendl MC, Kota K, Weinstock GM, Mitreva M. 2013. Coverage theories for metagenomic DNA sequencing based on a generalization of Stevens’ theorem. J Math Biol 67:1141–1161. doi:10.1007/s00285-012-0586-x PMC3795925 22965653

[12] Alneberg J, Bjarnason BS, de Bruijn I, Schirmer M, Quick J, Ijaz UZ, Lahti L, Loman NJ, Andersson AF, Quince C. 2014. Binning metagenomic contigs by coverage and composition. Nat Methods 11:1144–1146. doi:10.1038/nmeth.3103 25218180

